# Radiofrequency catheter ablation for paroxysmal supraventricular tachycardia in children: Insights on its safety and efficacy from a lower middle-income country

**DOI:** 10.7150/ijms.86594

**Published:** 2023-08-15

**Authors:** Bui The Dung, Nguyen Minh Nhut, Luong Cao Son, Bui Gio An, Nguyen Minh Duc, Dang Van Phuoc

**Affiliations:** 1 Department of Cardiology, University Medical Center HCMC, Ho Chi Minh City, Vietnam; 2 Department of Cardiology, Children's Hospital 1, Ho Chi Minh City, Vietnam; 3 Department of Radiology, Pham Ngoc Thach University of Medicine, Ho Chi Minh City, Vietnam; 4 School of Medicine, Vietnam National University, Ho Chi Minh City, Vietnam

**Keywords:** Radiofrequency catheter ablation, children, paroxysmal supraventricular tachycardia

## Abstract

**OBJECTIVE:** Radiofrequency catheter ablation (RFCA) is a safe and effective treatment for paroxysmal supraventricular tachycardia in adults. However, data on its use in children, particularly from low- and middle-income countries, are limited. This study aimed to evaluate the safety and efficacy of RFCA in children with paroxysmal supraventricular tachycardia from Vietnam.

**METHODS:** A prospective study was conducted from January 2009 to July 2016 at the University Medical Center, Ho Chi Minh City, Vietnam. Ninety-five children diagnosed with paroxysmal supraventricular tachycardia were enrolled; 90 patients underwent cardiac electrophysiology and RFCA. The patients were followed up for 3-12 months, with a mean follow-up period of 7.5 ± 2.3 months.

**RESULTS:** The average age of the patients was 10.5 ± 3 years, with the youngest patient being 4 years old; 46.3% of the patients were female and 53.7% were male. The patients' average weight was 35.2 ± 9.6 kg. Atrioventricular reentrant tachycardia accounted for 72.6% of the cases and atrioventricular nodal reentrant tachycardia for 27.4% of the cases; no patients had atrial tachycardia. The success rate of RFCA was 98.9% (89/90 patients). During the 12-month follow-up, 5.6% of the patients experienced recurrence but were successfully treated with a second ablation. No severe complications were reported during the procedure and follow-up.

**CONCLUSIONS:** This study found RFCA to be a safe and effective treatment for paroxysmal supraventricular tachycardia in children. It demonstrated a high success rate and low recurrence and complication rates for RFCA in children, thereby highlighting the potential advantages of the procedure as a treatment option.

## Introduction

Paroxysmal supraventricular tachycardia (PSVT), including atrioventricular reentrant tachycardia (AVRT), atrioventricular nodal reentrant tachycardia (AVNRT), and atrial tachycardia, is a common arrhythmia in children and presents with various clinical manifestations 1, 2. Symptoms range from mild indications, such as palpitations and anxiety, to severe manifestations, including heart failure, syncope, and even sudden death 3. These symptoms are influenced by age, heart rate, duration of tachycardia, and preexisting structural heart disease. Although antiarrhythmic drugs are often used for treatment, their efficacy is less than 70% and is accompanied by frequent side effects 2. In recent years, radiofrequency catheter ablation (RFCA) has emerged as a promising treatment for PSVT, with a high success rate and low complication rates 4. However, studies on RFCA in children with PSVT remain scarce in Vietnam. Therefore, this study aimed to investigate the electrophysiological characteristics of PSVT in children and assess the effectiveness and safety of RFCA after three to twelve months of follow-up.

## Methods

This prospective study was conducted between January 2009 and July 2016. It was approved by the Scientific Research Committee of the University Medical Center HCMC. Prior to participation, the children and their parents were provided with a detailed explanation of the procedure and they signed an informed consent form. No inducements or benefits were offered to the patients for enrolling in the study. The electrocardiogram, blood pressure, and peripheral capillary oxygen saturation were continuously monitored in the initial 24-hour postoperative period. The puncture site was regularly screened for hematoma. Before discharge, an electrocardiogram was recorded to identify potential atrioventricular block or recurrence.

### Inclusion Criteria

The criteria for inclusion in this study were that the patients were children under 16 years of age with a diagnosis of PSVT confirmed by electrophysiological (EP) study and that they were indicated for RFCA according to the North American Society of Pacing and Electrophysiology (NASPE) 2002 guidelines. Patients under the age of 5 years with a Class I indication for RFCA were also included.

### Exclusion Criteria

Patients with acute infection, coagulopathy, severe systemic disease, intracardiac thrombosis, or vascular malformations that would prevent catheter insertion were excluded.

### Electrophysiology Study

The pediatric patients were adequately prepared for EP study by ensuring that they adhered to guidelines for fasting for a minimum of six hours and hydration for a minimum of two hours prior to the procedure. In addition, antiarrhythmic medication was discontinued for at least five times the half-life of the drugs before the procedure. Anesthesia was induced with propofol.

During the procedure, two 4/5-Fr quadripolar electrophysiology catheters (St. Jude Medical, Little Canada, Minn, USA) were placed in right ventricle apex and His bundle, 5/6-Fr Livewire steerable electrophysiology catheter (St. Jude Medical, Little Canada, Minn, USA) was in coronary sinus. All of them were inserted via the femoral veins. Programmed electrical stimulation was employed to identify atrioventricular nodal conduction, accessory pathways, and induction and termination of tachycardia. An endocardial map was created to pinpoint the location of accessory pathways, the slow pathway, and foci of atrial arrhythmia for subsequent RFCA.

### Radiofrequency Catheter Ablation

The optional ablation mode was configured to a control temperature of 60 °C and energy of 30-50 W. To be effective, ablation was required to be prolonged for 60-120 s. In cases of RFCA of the left heart, patients were administered an additional dose 100U/kg of heparin to prevent thrombus formation. A four-pole catheter was used for ablation, with three diameters, 5F, 6F, and 7F, selected based on weight and location. Livewire™ TC Ablation Catheter (St. Jude Medical, Little Canada, Minn, USA) was selected to ablate. The catheters commonly used for ablation were 6F and 7F in size. C-curve/6.4-cm and D-curve/6.4-cm catheters were used to ablate the accessory pathway in the free wall, while A-curve/3.8-cm catheters were used for ablation in the left posterior septum. Continuous electrocardiogram monitoring was conducted during RFCA. Ablation was immediately discontinued if an atrioventricular block, junctional tachycardia, catheter displacement, a sudden increase in temperature or impedance, a bubble, or failure to raise temperature to near-controllable levels occurred.

The RFCA accessory pathway was considered successful when bidirectional conduction was blocked through the accessory pathways during atrial and ventricular stimulation. Similarly, slow-pathway ablation was considered successful when AVNRT episodes were no longer induced with programmed cardiac stimulation, with or without intravenous isoproterenol. The success criterion for atrial tachycardia was defined as its absence during introduction of extrastimuli and atrial burst pacing. These criteria were used to evaluate the effectiveness of RFCA in this study.

### Follow-Up

Patients who underwent left-sided accessory pathway ablation were prescribed aspirin at a dose of 3-5 mg/kg/day for 30 days post-discharge. The patients underwent electrocardiogram testing every month in the first three months following the procedure and then every three months or whenever symptoms indicated recurrence of tachycardia.

### Statistical Analysis

The descriptive statistics of quantitative variables are presented as mean ± standard deviation if they are normally distributed or median and interquartile range (25-75) if they are not normally distributed. The comparison of proportions between two groups was performed using the chi-square test or Fisher's exact test. Qualitative variables are described as frequency and percentage. An independent *t*-test was employed to compare the means of two groups, while ANOVA was used for comparisons of quantitative variables between three or more groups. The level of statistical significance was set at p < 0.05. All statistical analyses were performed using STATA 14 (Stata Corp, Texas, USA).

## Results

### Demographic Features

Ninety-five patients (46.3% female) fulfilling the inclusion criteria were enrolled in this study. Their average age was 10.5 ± 3 years (range 4-16 years). Five cases were excluded due to refusal to undergo RFCA after being counseled about the potential risk of atrioventricular block. The remaining 90 patients (98.9%) underwent RFCA, with only one case (1.1%) of fast-slow AVNRT providing unsuccessful. Only one patient was younger than 5 years of age, comprising 1.1% of the study population. The average follow-up period was 7.5 ± 2.3 months. The participants' average weight was 35.2 ± 9.6 kg. Four cases (4.2%) presented with congenital heart disease, including two cases of Ebstein's anomaly, one case of partial atrioventricular defect, and one case of single ventricle with left-sided morphology. All four cases exhibited tachycardia related to the atrioventricular accessory pathway, which was surgically corrected prior to ablation. The observed severe symptoms included hypotension (11.6%), syncope (5.3%), tachycardia requiring cardioversion (3.2%), and persistent tachycardia during EP study (5.3%).

### Electrophysiology Study and Radiofrequency Catheter Ablation

**Figure 1:**
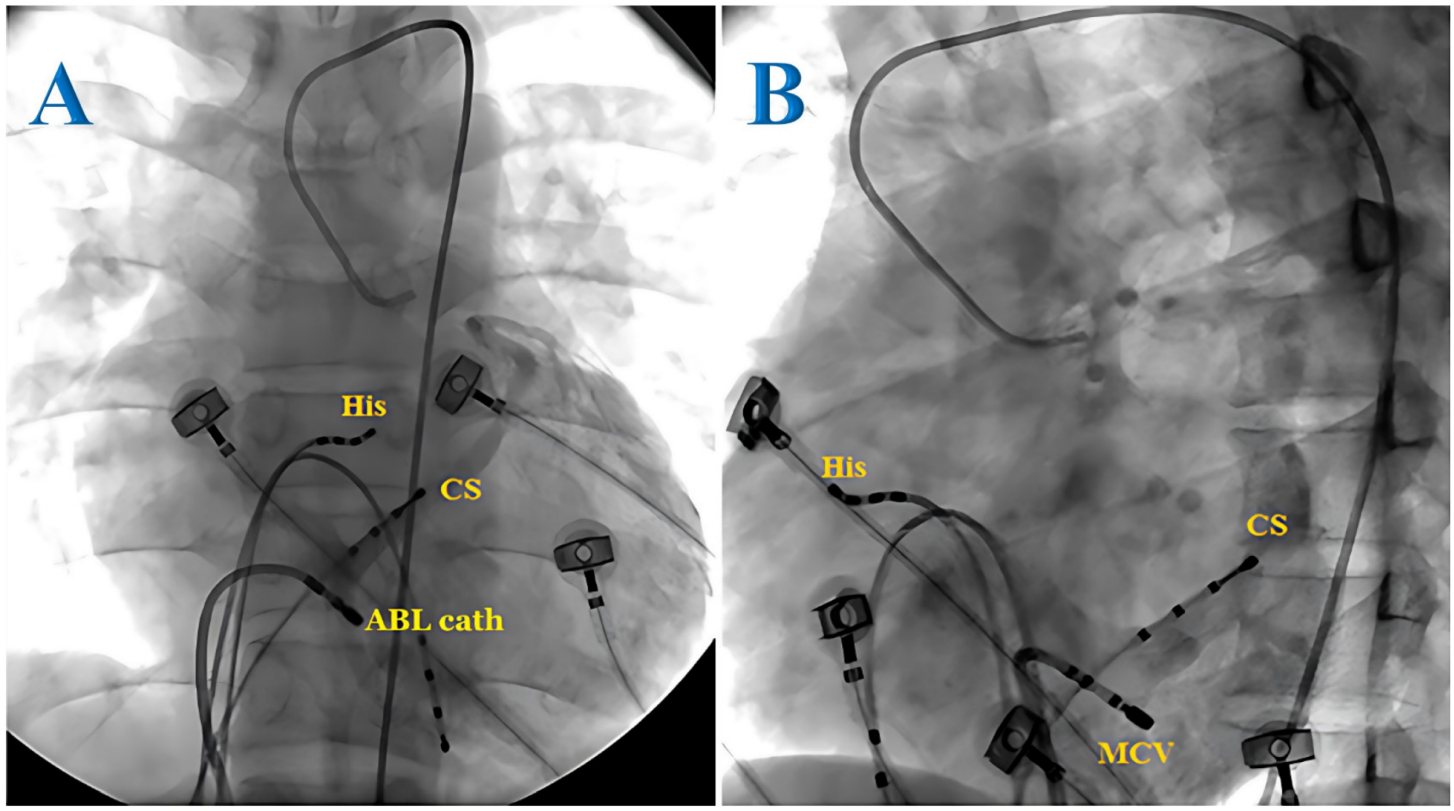
Radiofrequency ablation of the epicardial accessory pathway located in the coronary sinus of a pediatric patient from the anteroposterior view (A) and left anterior oblique view (B). ABL cath: ablation catheter; CS: coronary sinus; His: His bundle.

**Figure 2:**
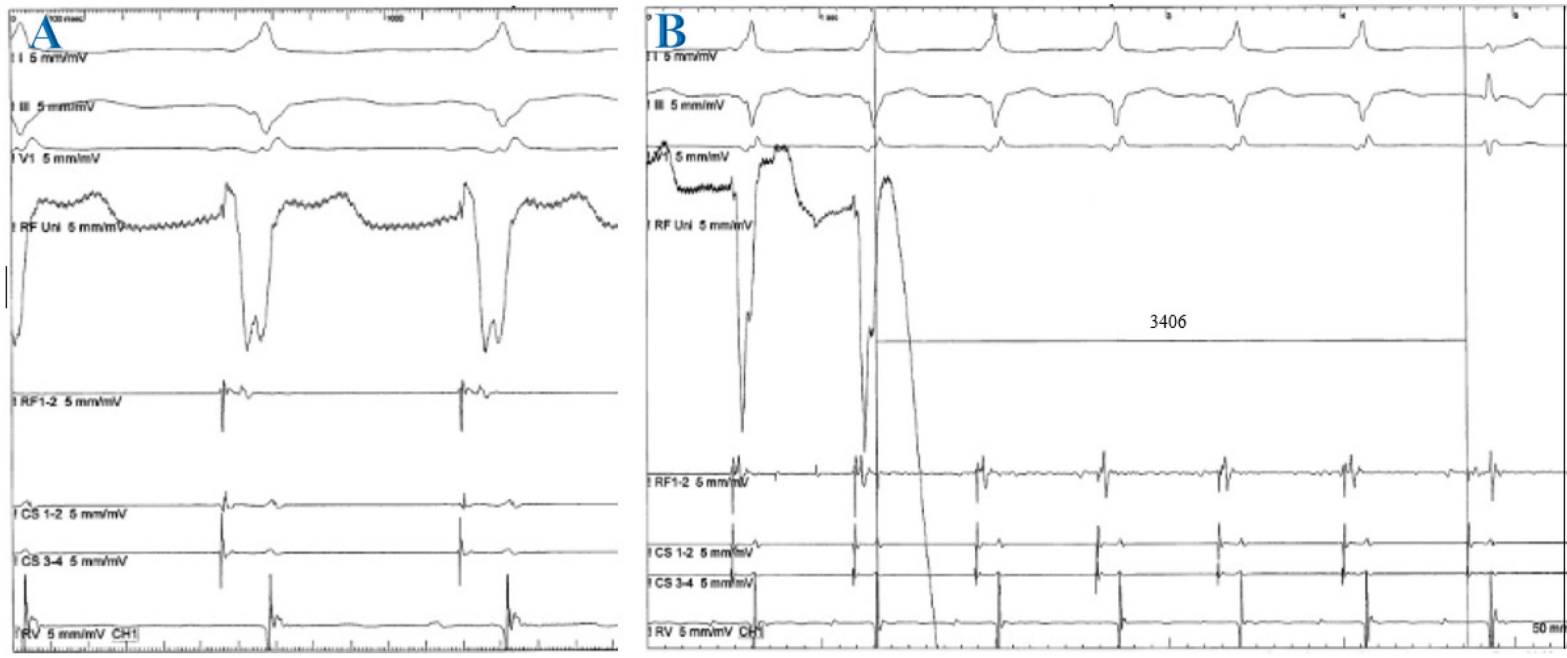
Surface electrocardiogram (lead I, III, V1) and intracardiac recordings from ablation catheter (RF), coronary sinus (CS) catheter and right ventricle (RV). CS 3-4 to 1-2: proximal to distal CS. **A**: *Ablation site.* **B**: *Elimination of the manifest accessory pathway after successful application of RF energy was given for 3406 ms*.

The tachycardia in most cases (72.6%) in this study was attributed to the atrioventricular accessory pathway, with 29.5% of the cases presenting with Wolff-Parkinson-White (WPW) syndrome and 43.1% of the cases with concealed accessory pathway. AVRT accounted for 27.4% of the cases and no cases of atrial tachycardia were observed. Of the 69 patients with tachycardia associated with accessory pathways, three cases (4.3%) presented with two accessory pathways. The most frequent accessory pathway locations were the left lateral (37.5%) and right lateral (23.6%) positions. In our study, 1 of 10 posterior wall accessory pathways was found to be located in the CS. In this case, after multiple unsuccessful endocardial ablation attempts in the posterior wall region, we decided to explore the CS and successfully ablated the accessory pathway. We then performed a retrograde CS angiogram and determined the ablation site to be in the middle cardiac vein (Figures 1 and 2). In patients with WPW syndrome, the most common site of the accessory pathway was the right side (33.3%), while the concealed accessory pathway was dominant in the left side (45.2%) (Table 1). Orthodromic AVRT was observed in 98.5% of the cases, with one patient showing antidromic AVRT (1.5%). The approach to the left-sided accessory pathways was through retrograde aortic access. No cases of pre-excited atrial fibrillation were detected. The average heart rate during tachycardia was 193 beats/min.

In the group of 26 patients diagnosed with AVNRT, 96.2% presented with the typical slow-fast form, while the remaining 3.8% exhibited the atypical fast-slow form. An AH jump was documented in 84.6% of the cases. The average heart rate during tachycardia was 192 beats/min. The AH interval in patients with AVNRT was significantly longer than in those with tachycardia associated with accessory pathways (Table 2).

During follow-up, recurrence was noted in 5.6% of patients, all of whom were successfully ablated for a second time. Ablation success and repetition were similar for AVNRT and accessory pathways (Table 3).

The number of RFCA applications required for successful ablation of manifest and concealed accessory pathways was comparable. In addition, the number of pulses needed for success in accessory pathway ablation did not differ from that required for AVNRT ablation. However, concealed accessory pathway ablation required longer fluoroscopy (p = 0.017) and procedure time (p = 0.009) than manifest accessory pathway ablation. Similarly, accessory pathway ablation required a more prolonged procedure and fluoroscopy time than slow pathway ablation in AVNRT.

No severe complications (death, atrioventricular block requiring pacemaker, tamponade, pneumothorax, and transfusion) were noted in cases that underwent RFCA. There were 7 cases with mild complications (7.7%). Among them, 4 patients experienced hematoma at the puncture sites, including three cases with arterial access for left side accessory pathways ablation and one case requiring a 9-Fr sheath to retrieve a entangled ablation catheter in the femoral vein. There were 2 patients (one case was mid-septal accessory pathway and the other was atypical AVNRT) had transient ventricular block during the ablation. And 1 case with 5-Fr ablation catheter was entrapped in the right iliac vein, as described below. These mild complications were promptly managed and did not result in any lasting sequelae (Table 4).

We had one case of catheter entrapment during maneuver. Patient was a 6 years-old boy with a weight of 20 kilograms and a height of 108 centimeters. During insertion a reused 5-Fr Livewire™ TC Ablation Catheter (St. Jude Medical, Little Canada, Minn, USA), the tip of catheter was entrapped in the right internal iliac vein, making it impossible to retract or advance further. The plastic casing of the reused catheter was cracked, broken, and entangled within the blood vessel wall, which was a reasonable cause for this issue. We attempted to retrieve the entangled catheter by cutting it externally and inserting a long 9-Fr sheath to facilitate its removal; however, this initial approach proved unsuccessful. Subsequently, we employed a 9-Fr sheath from the left femoral vein, advancing it carefully to the site of the entrapped catheter. With the use of a snare, we successfully captured the broken tip of the catheter and safely extracted it, achieving a successful retrieval.

## Discussion

Most patients in this study were aged between 5 and 16 years (98.9%), a demographic that aligns with the guidelines outlined by NASPE for ensuring patient safety during ablation procedures. It is important to note that ablation lesions can enlarge over time. Therefore, the procedure is typically only recommended for children under the age of 5 years who have WPW syndrome and have experienced resuscitated sudden death or refractory tachycardia causing ventricular dysfunction.

The age and gender distributions of participants in this study are similar to those in previous studies. For instance, in the multicenter COMPAS study, only 7.1% of children under the age of 5 years received tachycardia ablation 5. Moreover, the average weight of patients in our investigation was 35.2 ± 9.6 kg, comparable to that in Hafez *et al.*'s study 6, but was significantly lower than that in Joung *et al.*'s study 7. This discrepancy may be attributed to two factors: firstly, the mean age of patients in Joung et al.'s study was higher; and secondly, children in developed countries, such as Korea, tend to weigh more than those in developing countries like Vietnam and Egypt.

In this study, the prevalence of congenital heart disease was 4.2%, with Ebstein's anomaly being the most common. All patients with congenital heart disease had tachycardia associated with the atrioventricular accessory pathway, a phenomenon well documented in the literature 8, 9. These results are consistent with findings reported in previous studies. In this study, the majority of patients developed tachycardia during cardiac EP study, and a quarter of the patients experienced severe symptoms during tachycardia. However, 3 of the 26 patients with AVNRT required intravenous isoproterenol to induce tachycardia, as recommended by Stellbrink *et al.* 10.

The epicardial accessory pathways related to the coronary sinus (CS) occupy approximately 19-33% of the atrioventricular pathway and may be located in the CS ostium, proximal CS, or CS diverticulum 11. CS injury due to ablation should be considered, and, therefore, CS angiography is recommended. In Müller's study, the overall success rate of CS ablation was 90.9%, with a CS injury rate of 9%, and 23% of patients experienced recurrence of PSVT after an 8.4-year follow-up 12.

The occurrence of catheter entrapment is rare. Even in large centers, experiences in handling such cases have only been reported case by case. Joung *et al.* 7 described one case (0.8%) where a reusable catheter was trapped in the leaflet of the tricuspid valve. Chu *et al.* 13 also represented a case which a 7‐Fr steerable duodecapolar catheter (Livewire, Daig, St. Jude Medical) became entangled in the Chiari network. Experts suggest that the guiding sheath can be advanced over the catheter and the catheter can then be slowly withdrawn from within the sheath 14, 15.

The results of this study indicate that RFCA in children has few complications; the electrophysiologist's experience is a crucial factor in reducing complications. For high-risk accessory pathways, it is advisable to start with low energy and temperature or consider cryoablation to minimize the risk of complications. Overall, the low complication rate and high success rate of RFCA in treating pediatric patients with PSVT support its continued use as a safe and effective treatment.

At the present time, Vietnam's EP capabilities are developing and need strengthening with further research and lessons to learn. Therefore, multicenter studies with large samples and a wider age range are needed for a deeper understanding of EP for children in Vietnam. A limitation of this study was the relatively short follow-up time; therefore, extending the follow-up time is necessary to manage delayed recurrence.

## Conclusions

RFCA is a safe and effective therapeutic option for managing PSVT in children. It has consistently shown high success rates and low recurrence rates, with few complications. However, it is important to note that the electrophysiologist's experience of performing RFCA plays a critical role in determining the safety and efficacy of the procedure. In addition, for high-risk accessory pathways, cautious approaches, such as starting with low energy and temperature and cryoablation, are advisable. Further research and long-term follow-up studies are necessary to fully establish the effectiveness and safety of RFCA as a treatment for PSVT in the pediatric population.

## Figures and Tables

**Table 1:** Number and sites of manifest and concealed accessory pathways in pediatric patients with tachycardia attributed to the atrioventricular accessory pathway; some cases presented with Wolff-Parkinson-White (WPW) syndrome and others with concealed accessory pathways.

	WPW syndrome n (%)	Concealed accessory pathways n (%)
**Number of accessory pathways**		
1	26 (92.9)	40 (97.6)
2	2 (7.1)	1 (2.4)
**Site of accessory pathways**		
Left lateral	8 (26.7)	19 (45.2)
Right lateral	10 (33.3)	7 (16.7)
Right postero-septal	6 (20.0)	3 (7.1)
Right mid-septal	1 (3.3)	0 (0)
Right antero-septal	1 (3.3)	0 (0)
Para-Hisian	0 (0)	4 (9.5)
Left postero-septal	0 (0)	1 (2.4)
Left postero-lateral	0 (0)	4 (9.5)
Right postero-lateral	2 (6.7)	1 (2.4)
Right antero-lateral	2 (6.7)	0 (0)
Left antero-lateral	0 (0)	3 (7.1)

**Table 2:** Electrophysiology characteristics of two types of tachycardia, atrioventricular nodal reentrant tachycardia (AVNRT) and accessory pathway (AP)-mediated tachycardia, in pediatric patients.

	AVNRT	AP-mediated tachycardia	*p*
	Mean (SD)	Mean (SD)	
CL (ms)	314.5 (48.8)	315.9 (36.9)	0.876
QRS (ms)	80.8 (12.6)	78.5 (9.6)	0.360
AH (ms)	215.4 (40.2)	138.6 (31.6)	< 0.001
HV (ms)	38.4 (4.2)	36.5 (4.2)	0.059

**Table 3:** Results of radiofrequency catheter ablation in pediatric patients with Wolff-Parkinson-White (WPW) syndrome, concealed accessory pathways (AP), or atrioventricular nodal reentrant tachycardia (AVNRT).

	WPW*	Concealed AP**	p	AVNRT	p
Success, n (%)	27 (100)	37 (100)		25 (96.2)	0.289
Failure, n (%)	0 (0)	0 (0)		1 (3.8)	
Repeat, n (%)	2 (7.4)	1 (2.7)		2 (8.0)	0.603
Mean fluoroscopy time (min), mean (SD)	17 (8.7)	23.4 (11.2)	0.017	17 (8.7)	0.041
Mean procedure time (min), mean (SD)	95.4 (27.9)	115.9 (31.5)	0.009	95.4 (27.9)	0.026
Mean RF pulses, mean (SD)	3.9 (2.8)	5.0 (2.6)	0.102	4.3 (2.2)	NS

*One case refused ablation; **four cases refused ablation; NS: non-significant.

**Table 4:** Complications related to radiofrequency catheter ablation in pediatric patients.

	n	%
Hematoma*	4	4.4
Transient atrioventricular block**	2	2.2
Catheter entrapment	1	1.1
**Total**	**7**	**7.7**

## Abbreviations

AVNRT: Atrioventricular nodal reentrant tachycardia
AVRT: Atrioventricular reentrant tachycardia
EP: Electrophysiology
NASPE: North American Society of Pacing and Electrophysiology
PSVT: Paroxysmal supraventricular tachycardia
RFCA: Radiofrequency catheter ablation
WPW: Wolff-Parkinson-White
